# *miR-17* promotes expansion and adhesion of human cord blood CD34^+^ cells in vitro

**DOI:** 10.1186/s13287-015-0159-1

**Published:** 2015-09-07

**Authors:** Yuxia Yang, Saifeng Wang, Zhenchuan Miao, Wei Ma, Yanju Zhang, Li Su, Mengyu Hu, Junhua Zou, Yuxin Yin, Jianyuan Luo

**Affiliations:** Department of Medical & Research Technology, School of Medicine, University of Maryland, Baltimore, MD 21201 USA; Institute of Systems Biomedicine, Department of Pathology, School of Basic Medical Sciences, Peking University, Beijing, China; Beijing Vitalstar Biotechnology Co., Ltd., Beijing, China; Department of Histology and Embryology, School of Basic Medical Sciences, Capital Medical University, Beijing, China; Tianjin Central Hospital for Obstetrics and Gynecology, Tianjin, China; Center of Medical and Health Analysis, Peking University, Beijing, China; Department of Medical & Research Technology, Department of Pathology, School of Medicine, University of Maryland, College Park, USA

## Abstract

**Introduction:**

We have recently found that *miR-17* is necessary in the cell-extrinsic control of cord blood (CB) CD34^+^ cell function. Here, we demonstrated that the proper level of *miR-17* is also necessary in the cell-intrinsic control of the hematopoietic properties of CB CD34^+^ cells.

**Methods:**

The *miR-17* overexpression and knockdown models were created using primary CB CD34^+^ cells transfected by the indicated vectors. Long-term culture, colony forming, adhesion and trans-well migration assays were carried out to investigate the function of *miR-17* on CB CD34^+^ cells in vitro. NOD prkdc^*scid*^ Il2rg^*null*^ mice were used in a SCID repopulating cell assay to investigate the function of *miR-17* on CB CD34^+^ cells in vivo. A two-tailed Student’s *t*-test was used for statistical comparisons.

**Results:**

In vitro assays revealed that ectopic expression of *miR-17* promoted long-term expansion, especially in the colony-forming of CB CD34^+^ cells and CD34^+^CD38^−^ cells. Conversely, downregulation of *miR-17* inhibited the expansion of CB CD34^+^ cells. However, the overexpression of *miR-17* in vivo reduced the hematopoietic reconstitution potential of CB CD34^+^ cells compared to that of control cells. The increased expression of major adhesion molecules in *miR-17* overexpressed CB CD34^+^ cells suggests that the adhesion between *miR-17* overexpressed CB CD34^+^ cells and their niche in vivo is regulated abnormally, which may further lead to the reduced hematopoietic reconstitution capability of 17/OE cells in engrafted mice.

**Conclusion:**

We conclude that the proper expression of *miR-17* is required, at least partly, for normal hematopoietic stem cell–niche interaction and for the regulation of adult hematopoiesis.

**Electronic supplementary material:**

The online version of this article (doi:10.1186/s13287-015-0159-1) contains supplementary material, which is available to authorized users.

## Introduction

Hematopoiesis is a process capable of generating up to 300 million cells per minute in the bone marrow of an adult human [1]. All these cells arise from multipotent hematopoietic stem cells (HSCs) [2]. Continuous blood cell production for life is achieved by balancing self-renewal and differentiation among proliferating HSCs. This inner balance between self-renewal and lineage commitment is tightly controlled by integrating intrinsic and extrinsic mechanisms that govern the HSC state, which are still currently ambiguous [3–6].

MicroRNAs (miRNAs) are short non-coding RNAs (21 to 23 nucleotides in length) and are postulated to bind to 3′ untranslated regions of transcripts to post-transcriptionally regulate mRNA expression [7–9]. The important biological roles of miRNAs on hematopoiesis have been studied either by complete inactivation of miRNA formation or by selective targeting of specific miRNAs by many research groups. All of these studies suggest a major role for miRNAs in the regulation of hematopoietic cell commitment, proliferation, apoptosis, survival, and differentiation [10–13]. Recently, some miRNAs have been investigated in murine HSCs. Ectopic expression of *miR-181* in lineage negative hematopoietic progenitor cells from mouse bone marrow increased the fraction of B lineage cells (CD19^+^) in vitro and in vivo [14]. Enforced expression of *miR-29a* induced aberrant self-renewal in downstream progenitors, resulting in a low penetrant acute myeloid leukemia disease [15]. *miR-17-92* cluster increasing expands multipotent hematopoietic progenitors, while imbalanced expression of its individual oncogenic miRNAs promotes leukemia in mice [16]. *miR-125b* supports myelopoiesis but not granulocyte colony-stimulating factor-induced granulocytic differentiation, and enforced expression of *miR-125b* induced an initial myeloproliferative disorder depending upon the ectopic expression levels [17–19]. Collectively, these studies indicate that miRNAs may be important regulators of hematopoiesis.

*miR-17* (also called *miR-17-5p*), an important member of the *miR-17-92* cluster, contains the AAAGUGC-seed sequence [20]. *miR-17* is abundantly expressed in murine hematopoietic progenitors and increased expression of AAAGUGC-seed containing miRNA in lineage negative bone marrow cells promotes replating capacity and expansion of myeloid progenitors [21]. However, according to the model for HSC/hematopoietic progenitor cell (HPC)-expressed miRNA-mediated control of human hematopoiesis predicted by Georgantas et al*.*, ectopic expression of *miR-17* in peripheral blood cells may inhibit both myeloid and erythroid colony growth [22]. Fontana et al. reported that downregulation of the *miR-17-92* cluster can promote myeloid lineage fate, which is in line with the prediction of Georgantas et al. [23]. Moreover, Li et al. showed that the *miR-92a*-induced erythroleukemia cell line, when overexpressing *miR-17*, displayed a significantly reduced proliferation rate, exhibited morphological features of apoptosis, and ultimately died 2 weeks post-transduction [16]. Although these studies all indicate that *miR-17* is an important regulator of hematopoiesis, the function of *miR-17* on hematopoiesis remains controversial. Moreover, most of the data about *miR-17* to date were obtained from murine studies while the relevance to human HSC still needs to be substantiated.

Recently, we have found that *miR-17* is necessary in the cell-extrinsic control of HSC and HPC function, which is, at least in part, through the augmented *HIF-1α* signal pathways in osteoblasts [24]. Here, we reported that *miR-17* is also necessary in the cell-intrinsic control of governing the biological properties of human cord blood (CB) CD34^+^ cells in vitro and in vivo. Our data showed that *miR-17* is significantly expressed in human CB CD34^+^CD38^−^ cells compared to the levels expressed in the CD34^+^CD38^+^ cells or mononuclear cells (MNCs). By overexpression and knockdown studies, we showed that ectopic expression of *miR-17* promotes long-term expansion and colony forming of CB CD34^+^ cells and CD34^+^CD38^−^ cells in vitro. Knockdown of *miR-17*, on the other hand, resulted in decreased CB CD34^+^ cell expansion, which consequently diminished the cell number. However, it is to be noted that the hematopoietic reconstitution potential of *miR-17*-overexpressed CB CD34^+^ cells in vivo were significantly reduced in contrast with that of control CB CD34^+^ cells in repopulating assays with NPG mice. We further found that the expression of selected major adhesion molecules on CB CD34^+^ cells was increased and the specific adhesion of these cells to N-cadherin and vascular cell adhesion molecule-1 (VCAM1) were also enhanced upon *miR-17* overexpression in vitro. The adhesion potential of 17/OE CB CD34^+^ cells to VCAM1 was significantly reduced following β_1_-integrin knockdown, which suggested that β_1_-integrin expressed on 17/OE CD34^+^ cells mediated, at least in part, the increase in interaction between 17/OE CD34^+^ cells and VCAM1 caused by ectopic *miR-17*. Our data imply that the adhesion between *miR-17*-overexpressed CB CD34^+^ cells and their niche in vivo is regulated abnormally, which may further lead to the reduced hematopoietic reconstitution capability of 17/OE cells in engrafted mice.

## Materials and methods

### Cell cultures

Human CB samples were obtained as described previously [24, 25]. After isolation by lymphocyte separation medium (TBD Biotech, Tianjing, China), CB MNCs were immunomagnetically enriched for CD34^+^ cells using the MACS CD34^+^ Progenitor Cell Isolation Kit (Miltenyi Biotech Inc., Bergisch Gladbach, Germany) according to the manufacturer’s instructions. The purity of CD34^+^ cells was about 80–90 %, as determined by flow cytometry. CB CD34^+^ cells were resuspended and stained with CD38 (Miltenyi Biotech Inc.) in phosphate-buffered saline with 2 % fetal calf serum to obtain CD34^+^CD38^−^/CD38^+^ cells. Subsequent expansion of CD34^+^ cells or CD34^+^CD38^−^/CD38^+^ cells (1.0 × 10^4^/well) was carried out in StemSpan SFEM II (Stem Cell Technologies, Vancouver, BC, Canada), supplemented with 2 mM L-glutamine, 100 U/ml penicillin/streptomycin and a cytokine cocktail consisting of Flt ligand (25 ng/ml), SCF (25 ng/ml), thrombopoietin (25 ng/ml), and interleukin-6 (25 ng/ml) (Peprotech Inc., NJ, USA). After the indicated days of culture, the cells were collected by gentle pipetting from the culture and were used for further assessment. This study was approved by the Ethics Committee of Peking University. Before the experiments, the subjects were informed of the objectives, requirements and procedures of the experiments. All subjects gave informed written consent to participate in the study.

### Vector generation and transfection

The two *miR-17*-specific small hairpin RNAs (17/KD and 17/KD1) [24] and *β1-integrin*-specific shRNA (β1/KD) oligomers [26] were tested. Briefly, double-stranded DNA oligonucleotides were chemically synthesized and inserted into the pSUPER vector. Inserts were confirmed by sequencing. pSUPER vectors containing green fluorescent protein (GFP) and the indicated inserts were transfected into CD34^+^ cells by Lipofectanine 2000 (Invitrogen, CA, USA) following the manufacturer’s instructions. If not otherwise mentioned, pSUPER vectors containing scrambled target sequences not complementary to any known miRNA were served as controls for 17/KD (CTRL).

The human *pre-miR-17* gene was amplified by polymerase chain reaction (PCR) and subcloned into the vector pCMV-GFP to generate the expression constructs pCMV-GFP*-pre-miR-17* (17/OE). Vectors containing the empty intron sequence served as controls for 17/OE (CTRL). Isolated CD34^+^ cells were cultured in Stem-Span medium with the same supplements and cytokines as mentioned above. After 12–18 hours of culture, cells were transfected with the vectors. After FACS sorting with the FITC channel, the proportion of GFP-positive CD34^+^ cells reached 90 % 48 hours after transfection.

### RNA extraction and real-time PCR

Total RNA was extracted using the total RNA kit I (OMEGA, GA, USA). The first-strand cDNA was synthesized by the miRcute miRNA First-Strand cDNA Synthesis Kit (TIANGEN, BJ, China). Real-time PCR analysis was performed by ABI7500 (Applied Biosystems, CA, USA) using the miRcute miRNA qPCR Detection Kit (SYBR Green; TIANGEN). U6 snRNA (for miRNA) was used as the endogenous control. All samples were done in triplicate. The relative expression was determined using the ΔΔC_T_ method.

### Colony forming cell assay

Human colony forming cell assays were performed at day 7 or 14 of culture in a MethoCult GF H4434 “Complete” Methylcellulose medium with recombinant cytokines from Stem Cell Technologies. After 14–16 days of culture, colony-forming units (CFU-Cs) and lineage-specific colonies including colony-forming unit-erythrocyte (BFU-Es), colony-forming unit-granulocyte-macrophages (CFU-GMs), and colony-forming unit-mix (CFU-Mix’s), were counted under an inverted microscope and identified according to the manufacturer’s instructions.

### Antibodies

The following antihuman antibodies were used for flow cytometric analysis or sorting: CD38-APC (allophycocyanin), CD34-PE (phycoerythrin), N-cadherin-APC, β_1_-integrin-APC, CD44-APC, CXCR4-APC and CD49-APC (obtained from BD Biosciences, CA, USA). Each sample was blocked with 0.1 % bovine serum albumin in phosphate-buffered saline before staining. APC- or PE-conjugated mouse IgG1 (BD Biosciences) were used as isotype controls. Blocking antibody to CD29 (VLA4) was purchased from R&D Systems (Minneapolis, MN, USA). The anti-N-cadherin (GC-4) antibody was purchased from Sigma (St Louis, MO, USA). All antibody blocking treatments and staining were carried out for 40 minutes on ice.

### SCID repopulating cell assay

SCID repopulating cell assay was carried out as described previously [24]. Briefly, CB CD34^+^ cells were transfected, harvested as described above, and injected intravenously into 8-week-old, sublethally irradiated (1.8 Gy) NOD prkdc^*scid*^ Il2rg^*null*^ (NPG™, Vitalstar, Beijing, China) mice. The mice transplanted with freshly isolated CD34^+^ cells were used as positive controls. The mice were temporally monitored for 20 weeks and the peripheral blood (PB) was obtained through nonlethal eyebleeds under anesthesia with isoflurane as per institutional guidelines every 4 weeks. The mice were sacrificed at 20 weeks. Bone marrow MNCs were harvested and analyzed by flow cytometry. Human cell reconstitution was assessed using CD45, CD34, CD36, and GPA markers. Mouse IgG1 conjugated to PE, APC or PC7 were used as isotype controls. PCR amplification of the human 17α-satellite gene was employed as a second test for the presence of human cells in the NPG mice that had received transplants. All care and handling of animals was performed with the approval of the Institutional Authority for Laboratory Animal Care of Peking University.

### Adhesion and trans-well migration assays

Adhesion assay was carried out in a 48-well plate [27, 28]. Prior to use, wells were coated with ligands (N-cadherin 5 μg/ml, VCAM1 10 μg/ml) at 4 °C overnight. Supernatant was aspirated and wells were then incubated with 1 % bovine serum albumin for 30 minutes. Following incubation, CB CD34^+^ cells (5.0 × 10^4^ cells/well), resuspended in IMDM containing 2 % fetal calf serum, were added in each well and incubated for 90 minutes at 37 °C. After removing the nonadhering cells, the number of adhering hematopoietic cells was determined by colony-forming cell (CFC) assays at 37 °C with 5 % CO_2_ after 14–16 days of culture per experiment. Corresponding controls were also preformed for each condition.

Chemotactic responses of CD34^+^ cells to stromal derived factor 1 (SDF1α) were assessed in vitro by trans-well migration assay [28]. Briefly, 1.0 × 10^5^ of CD34^+^ cells were plated into the upper chamber of the transwell. SDF1α (100 ng) was added to the lower wells containing 600 μl of medium. The bubbles in the wells were removed gently. For each experimental set, some wells were kept without SDF1α to detect the spontaneous migration. The cells were allowed to migrate for 5 hours at 37 °C. After removing the upper wells, the migrated cells in the lower wells were collected and manually counted.

### Statistical analysis

The results are expressed as the mean ± standard deviation (SD). Statistical comparisons were performed using a two-tailed Student’s *t*-test.

## Results

### The expression levels of *miR-17* in CD34^+^CD38^−^ cells are higher than those in CD34^+^CD38^+^ cells

**Fig. 1:**
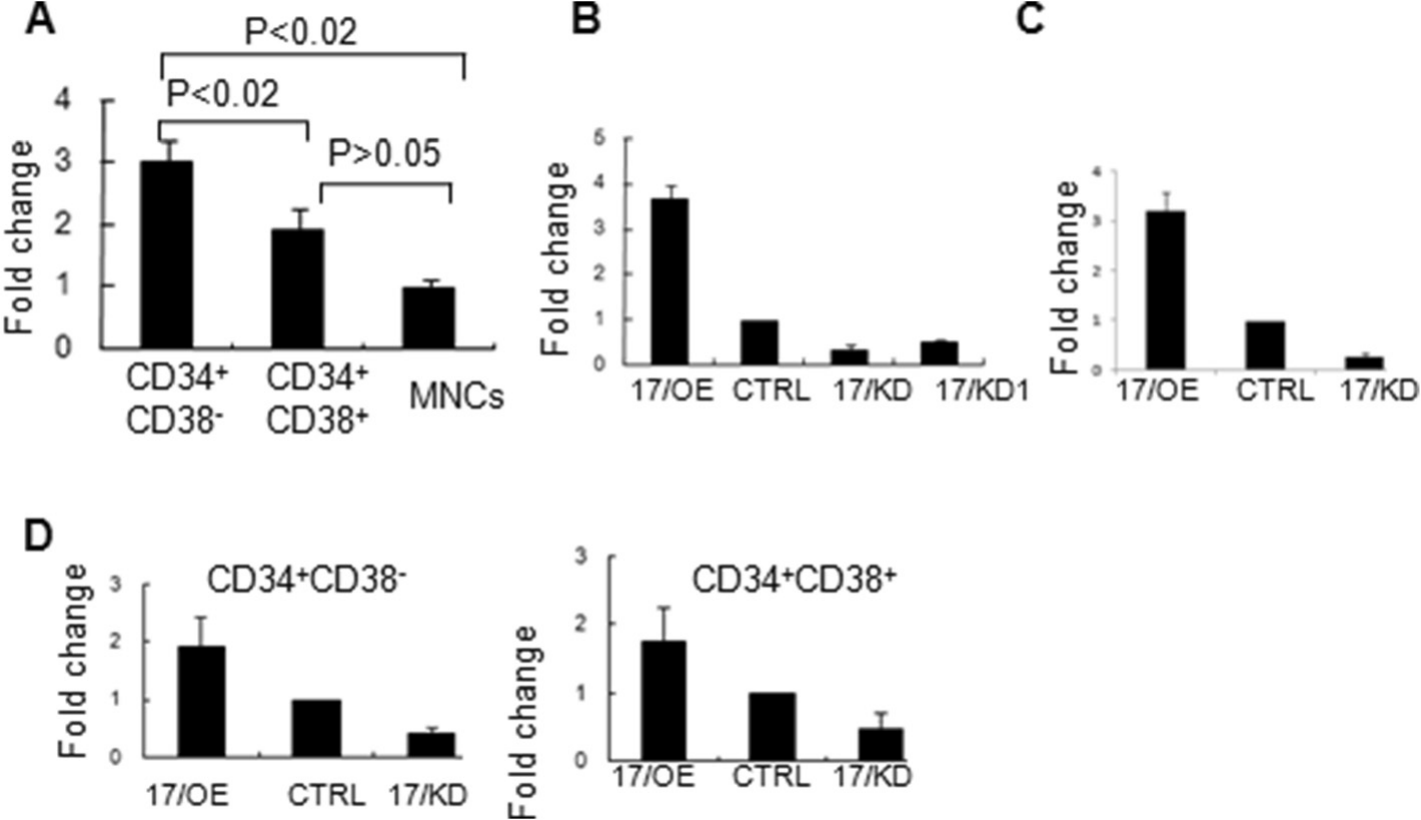
The expression of *miR-17* in CB hematopoietic CD34^+^CD38^−^/CD38^+^ cells. **a** The expression level of *miR-17* in CB CD34^+^CD38^−^/CD38^+^ cells was evaluated by real-time PCR. Each reaction was performed in triplicate. Student’s *t*-test was used for the statistical analysis between the two indicated populations. **b** Real-time PCR was performed to evaluate the expression level of *miR-17* in CB CD34^+^ cells after transfection with vectors for *miR-17* overexpression (17/OE), *miR-17* knockdown (17/KD or 17/KD1), or control (CTRL). The data are presented as the ratio of *miR-17* levels (relative to U6) in 17/OE, 17/KD or 17/KD1 to that in CTRL. Each reaction was performed in triplicate. **c** The expression level of *miR-17* in 17/OE CD34^+^ cells or 17/KD CD34^+^ cells 5 days after sorting was evaluated by real-time PCR. **d** The expression level of *miR-17* in 17/OE CD34^+^CD38^−^/CD38^+^ cells or 17/KD CD34^+^CD38^−^/CD38^+^ cells 20 days after culture was evaluated by real-time PCR

To determine the expression level of *miR-17* in human hematopoietic cells, we first obtained two populations from human CB MNCs through the analyses of human lineage-specific CD markers. The CD34^+^CD38^−^ populations expressed significantly higher levels of *miR-17* in comparison to those of the CD34^+^CD38^+^ populations or MNCs (Fig. 1a). The *miR-17* levels of the CD34^+^CD38^+^ populations showed a tendency, albeit insignificant, to be higher than those of the MNCs. The expression pattern of *miR-17* in human hematopoietic cells is consistent with that in 32D-CSF3R cells [21]. Collectively, *miR-17* levels are downregulated during the differentiation of human hematopoietic cells, which suggests that *miR-17* may play a role in regulating HSC function. To analyze the function of *miR-17* on primitive human hematopoietic cells, the *miR-17* overexpression (17/OE) and knockdown (17/KD or 17/KD1) models were created using primary CB CD34^+^ cells. The expression levels of *miR-17* were up- or downregulated in 17/OE and 17/KD, respectively (Fig. 1b) compared to those in CTRL. The levels of overexpression or knockdown 5 days or 20 days after culture in 17/OE CD34^+^CD38^−^/CD38^+^ cells or 17/KD CD34^+^CD38^−^/CD38^+^ cells were also checked, which maintained similar modulation (Fig. 1c and d). Based on shRNA influence on *miR-17* expression in CB CD34^+^ cells, 17/KD was chosen for further studies.

The transfection efficiency of vectors containing the *pre-miR-17* gene or shRNA specific for *miR-17* in CB CD34^+^ cells was measured by the expression of GFP (Additional file 1: Figure S1), which were evaluated under inverted fluorescence microscopy. Transfection in CB CD34^+^ cells was positive 24 hours after transfection. The proportion of GFP-positive CB CD34^+^ cells after FACS sorted with FITC channel reached 90 % 48 hours after transfection.

### *miR-17* promotes expansion and colony forming of CB CD34^+^ cells

**Fig. 2:**
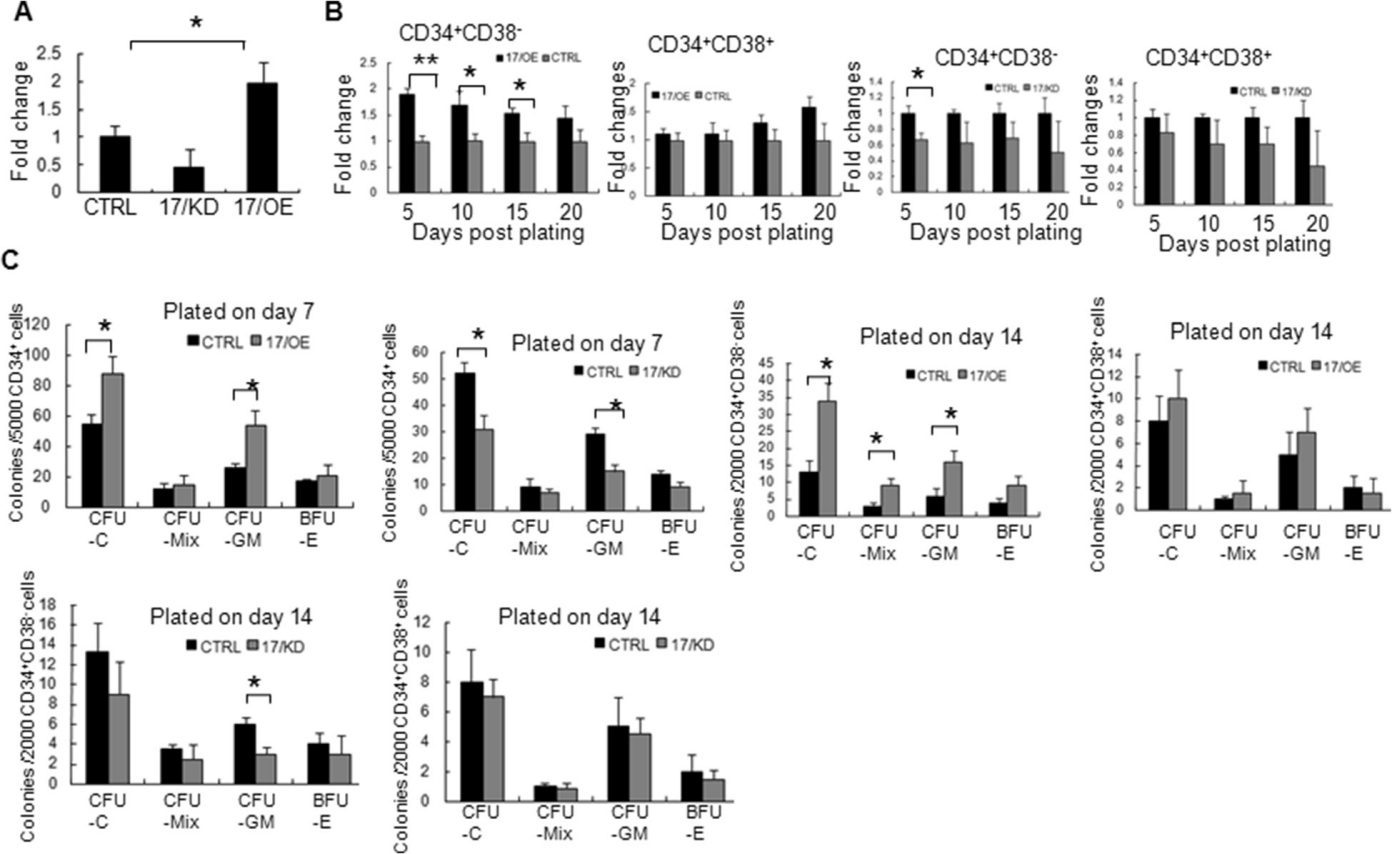
The effect of *miR-17* modulation on the expansion of CB CD34^+^CD38^−^/CD38^+^ cells. **a** The expansion of CB CD34^+^ cells upon *miR-17* modulation after culturing for 20 days. CB CD34^+^ cells were transfected with vectors for *miR-17* overexpression (17/OE), *miR-17* knockdown (17/KD), or control (CTRL) and cultured in cytokine-driven serum-free medium. Fold expansion is shown as mean + SD (n = 6). *p < 0.05, between 17/OE or 17/KD, and CTRL group (Student’s *t*-test). **b** CB CD34^+^ cells, transfected with ectopic *miR-17* vector (17/OE or 17/KD), were further separated through the analysis of CD38 expression. Cells were cultured in cytokine-driven serum-free medium and the cell numbers were monitored. The data are presented as the fold expansion (n = 6, mean + SD). *p < 0.05; **p < 0.01, between 17/OE or 17/KD and CTRL group (Student’s *t*-test). **c** Colony forming assays of CB CD34^+^ cells upon *miR-17* modulation. After 7 or 14 days of culture, the indicated cell numbers were plated for colony forming assays. The colonies, including BFU-Es, CFU-GMs, and CFU-Mix’s, with greater than 50 cells were counted after 14–16 days. The results are expressed as mean + SD (n = 6). *p < 0.05, between 17/OE or 17/KD, and CTRL group (Student’s *t*-test). *CFU* colony forming unit

We then investigated the ability of *miR-17* modulation to expand CB CD34^+^ cells in vitro. CD34^+^ cells were cultured in cytokine-driven serum-free medium. After 20 days of culturing, the number of total cells was counted. As shown in Fig. 2a, CD34^+^ cells were expanded significantly upon ectopic *miR-17*. After knockdown of *miR-17*, there was a trend toward a decrease in the total number of CD34^+^ cells, although statistical analyses of the cohort indicated that it did not meet statistical significance (*p* > 0.05). In order to assess whether there is a different response between CD34^+^CD38^−^ populations and CD34^+^CD38^+^ populations upon *miR-17* modulation, the expansion curves from the two populations from CD34^+^ cells were investigated. As shown in Fig. 2b, although both CD34^+^CD38^−^ and CD34^+^CD38^+^ cells were expanded to different extents upon *miR-17* modulation, the ectopic *miR-17* tends to expand CD34^+^CD38^−^ cells more, rather than CD34^+^CD38^+^ cells, especially during the first 15 days. The expansion fold of CD34^+^CD38^−^ cells from the 17/OE cells after being cultured for 15 days was significantly higher than that from the CTRL cells, especially during the first 5 days. Despite the presence of the HSC-supporting cytokines, CD34^+^CD38^−^ cells may undergo differentiation and become more committed along with culturing *in vitro*. Ectopic *miR-17* in CD34^+^ cells preferentially supports a specific expansion of the CD34^+^CD38^−^ populations, especially during the first 5 days, suggesting that the ectopic *miR-17* may specifically affect the least differentiated cells that have the characteristics of stem or early progenitor cells. After knockdown of *miR-17*, there was a trend toward a decrease in the expansion capacity of CD34^+^CD38^−^/CD38^+^ cells, although statistical analyses of the cohort indicated that it did not meet statistical significance (*p* > 0.05) outside of the first 5 days from CD34^+^CD38^−^ cells. Taken together, these data indicate that the ectopic *miR-17* in CD34^+^ cells preferentially supports a specific expansion of the CD34^+^CD38^−^ populations in vitro.

To investigate whether the expanded CB CD34^+^ cells after *miR-17* modulation maintained multipotent differentiation of HSC, we performed colony forming assays. CB CD34^+^ or CD34^+^CD38^−^ cells after transduction were cultured for 7 or 14 days in cytokine-driven serum-free medium and then subject to a CFU assay. After 14–16 days of culture, the colonies with greater than 50 cells were counted under an inverted microscope (Additional file 1: Figure S1). As shown in Fig. 2c, the number of total CFUs and CFU-GM from the 17/OE cells after being cultured for 7 days in vitro was significantly higher than that from CTRL. The number of CFU-Mix and BFU-E did not change significantly after *miR-17* overexpression. Knockdown of *miR-17* in CD34^+^ cells, on the other hand, resulted in reduced hematopoietic multipotential, which was followed by significantly diminishing CFC and CFU-GM output (Fig. 2c). We further examined the multipotent differentiation of CD34^+^CD38^−^/CD38^+^ cells upon *miR-17* modulation after cultured for 14 days in vitro. Compared with that from CD34^+^ cells after culturing for 7 days, there was a significant increase in the number of CFU-Mix from CD34^+^CD38^−^ cells upon *miR-17* overexpression (Fig. 2c). After knockdown of *miR-17*, there was a trend toward a decrease in the colony forming capacity of CD34^+^CD38^−^/CD38^+^ cells, although statistical analyses of the cohort indicated that it did not meet statistical significance (*p* > 0.05) outside of the CFU-GM forming capacity from CD34^+^CD38^−^ cells. Together, our data suggest that *miR-17* in CD34^+^ cells preferentially promotes a specific expansion of the CB CD34^+^CD38^−^ populations in vitro and the expanded CD34^+^CD38^−^ cells could differentiate into all of the lineages tested.

### Effects of *miR-17* modulation on the hematopoietic reconstitution capability of CB CD34^+^ cells in NPG mice

**Fig. 3:**
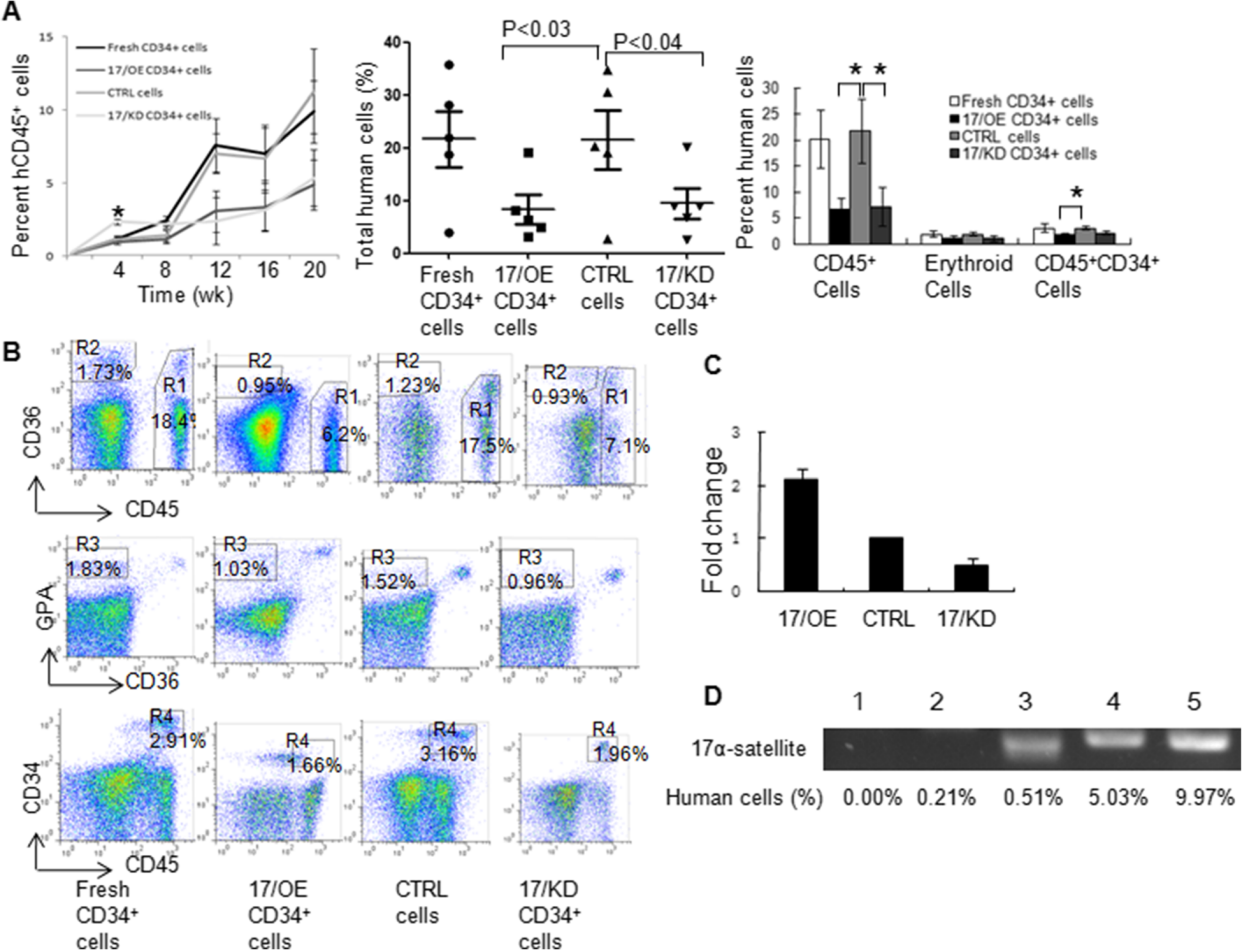
Effect of *miR-17* modulation on the hematopoietic reconstitution potential of CB CD34^+^ cells in NPG mice. **a** Effect of *miR-17* modulation on repopulation of CB CD34^+^ cells in NOD prkdc^*scid*^ Il2rg^*null*^ (NPG™) mice. 4.0 × 10^4^ *miR-17* overexpression (17/OE), *miR-17* knockdown (17/KD), or control (CTRL) CB CD34^+^ cells were injected intravenously into the sublethally irradiated NPG mice (n = 6 per group). The PB of NPG recipients was temporally monitored by analyzing CD45^+^ population every 4 weeks. The mice were sacrificed at 20 weeks after transplantation and the mononuclear cells from bone marrow were analyzed for human cells composed of CD45^+^, CD45^−^CD36^+^ and CD36^−^GPA^+^ cells and CD45^+^CD34^+^ population by flow cytometry. The level of total human cell engraftment is shown in the left panel. *p* < 0.03 or 0.04, between the NPG mice injected with 17/OE or 17/KD CD34^+^ cells and those injected with CTRL cells (Student’s *t*-test). The fraction of CD45^+^, erythroid (CD45^−^CD36^+^ and CD36^−^GPA^+^) and CD45^+^CD34^+^ population among the engrafted human cells is shown in the middle panel. The percent of CD45^+^ cells of PB from NPG recipients was shown in the right panel. *p < 0.05, between the NPG mice injected with 17/OE or 17/KD CD34^+^ cells and those injected with CTRL cells (Student’s *t*-test). The significant difference was analyzed between the mice injected with 17/OE or 17/KD CD34^+^ cells and those injected with CTRL cells. **b** Flow cytometry analysis of the human CB CD34^+^ cell repopulation in a representative NPG mouse after *miR-17* modulation. Fresh CD34^+^ cells served as controls. The MNCs from bone marrow harvested from the engrafted NPG mice were examined by flow cytometry for the assessment of human cells composed of CD45^+^ cells (R1), erythroid cells including CD45^−^CD36^+^ (R2) and CD36^−^GPA^+^ (R3) populations and CD45^+^CD34^+^ cells (R4). **c** The levels of *miR-17* expression in GFP-positive cells from engrafted mice at 20 weeks were tested by real-time PCR. **d** The bone marrow MNCs containing the different percentage of human cells (lanes 2–5) from the representative engrafted mice were analyzed for human-specific 17α-satellite DNA by PCR. The human-specific 17α-satellite gene was detected when the human cells were over 0.50 % (lanes 3–5) whereas it was indetectable at a percentage of 0.21 % (lane 2). Lane 1, one mouse without transplants; lane 2, one mouse receiving transplants of 17/OE CD34^+^ cells; lane 3, one mouse receiving transplants of 17/KD CD34^+^ cells; lane 4, one mouse receiving transplants of CTRL CD34^+^ cells; lane 5, one mouse receiving transplants of fresh CD34^+^ cells

To further support our in vitro expansion results, we examined the hematopoietic reconstitution potential of CB CD34^+^ cells after *miR-17* modulation in NPG mice. The sublethally irradiated NPG mice were transplanted with 4.0 × 10^4^ CB 17/OE CD34^+^ cells, CTRL, or CB 17/KD CD34^+^ cells using intravenous injection. We temporally monitored the PB of NPG recipients transplanted with *miR-17*-modulated CB CD34^+^ cells for 20 weeks by analyzing the percentage of human CD45^+^ cells using flow cytometry every 4 weeks. Although the percentage of human CD45^+^ cells gradually increased in the PB from all of the mice transplanted with *miR-17*-modulated CB CD34^+^ cells, the PB from 17/KD recipients displayed a significantly higher percentage of human CD45^+^ cells at 4 weeks than CTRL recipients, indicating that the hematopoietic reconstitution potential of 17/KD CD34^+^ cells is higher than that of CTRL CD34^+^ cells during the first 4 weeks (Fig. 3a left panel). The percentage of human CD45^+^ cells in the PB from the 17/OE or 17/KD group showed a tendency, although insignificant, to be lower than that from the CTRL group at 20 weeks after transplantation. The levels of total human cell engraftment composed of CD45^+^, CD45^−^CD36^+^ and CD36^−^GPA^+^ cells, and CD45^+^CD34^+^ populations were assessed in the bone marrow MNCs of the engrafted mice at 20 weeks post-transplant using flow cytometry. At 20 weeks, the amount of total human cells in the mice injected with 17/OE CD34^+^ cells or 17/KD CD34^+^ cells was significantly lower than that of the mice injected with CTRL CD34^+^ cells (Fig. 3a middle panel; *p* < 0.03 and 0.04, respectively), which indicates that the hematopoietic reconstitution ability of 17/OE CD34^+^ cells and 17/KD CD34^+^ cells is reduced in comparison with that of CTRL CD34^+^ cells. The statistical analysis was performed by comparing the mice injected with 17/OE or 17/KD CD34^+^ cells to those injected with CTRL cells. We further analyzed the multilineage development from the input CB CD34^+^ cells. Flow cytometry analysis of the human graft in a representative engrafted mouse from each group is shown in Fig. 3b. There was no significant difference in the percentage of erythroid cells, including CD45^−^CD36^+^ and CD36^−^GPA^+^ populations upon comparing the 17/OE or 17/KD group with the corresponding CTRL group (Fig. 3a right panel). However, we observed a significantly lower percentage of CD45^+^ cells in the 17/OE group compared to that in the CTRL group. Similarly, in contrast to the CTRL group, the 17/OE group also showed a significantly lower percentage of CD45^+^CD34^+^ cells (Fig. 3a right panel). The percentage of CD45^+^CD34^+^ cells in the 17/KD group whose transplants of CD34^+^ cells with *miR-17* knockdown showed a tendency, although insignificant, to be lower than that of mice receiving transplants of CTRL CD34^+^ cells. The observed multilineage development from input 17/KD CD34^+^ populations partly coincides with the results of expansion and colony forming assays in vitro. However, when the input cell numbers were similar, the 17/OE CD34^+^ cells contributed significantly less to hematopoietic reconstitution in recipient mice as opposed to the CTRL CD34^+^ cells, which was different from the in vitro expansion and colony forming assay, suggesting that the hematopoietic reconstitution capability of *miR-17*-overexpressed CB CD34^+^ cells were reduced in vivo. To confirm whether this inconsistency resulted from the change of *miR-17* expression in vivo, we checked the levels of *miR-17* expression in GFP-positive cells from engrafted mice at 20 weeks. As shown in Fig. 3c, the expression levels of *miR-17* were still up- or downmodulated in GFP-positive cells from NPG recipients transplanted with CB 17/OE CD34^+^ cells or 17/KD CD34^+^ cells, respectively, although the expression levels of *miR-17* became somewhat lower compared to that of corresponding initial cells. To further confirm that the human cells determined by flow cytometry were of human origin, the human-specific 17α-satellite gene was detected using PCR in several representative engrafted mice, which contained different percentages of human cells. We found that the human 17α-satellite gene could be detected by PCR amplification when the percentage of human cells was over 0.50 % (Fig. 3d, lanes 3–5), whereas it was undetectable at a percentage of 0.21 % (Fig. 3d, lane 2).

### The expression of adhesion molecules on CB CD34^+^ cells upon *miR-17* modulation

**Fig. 4:**
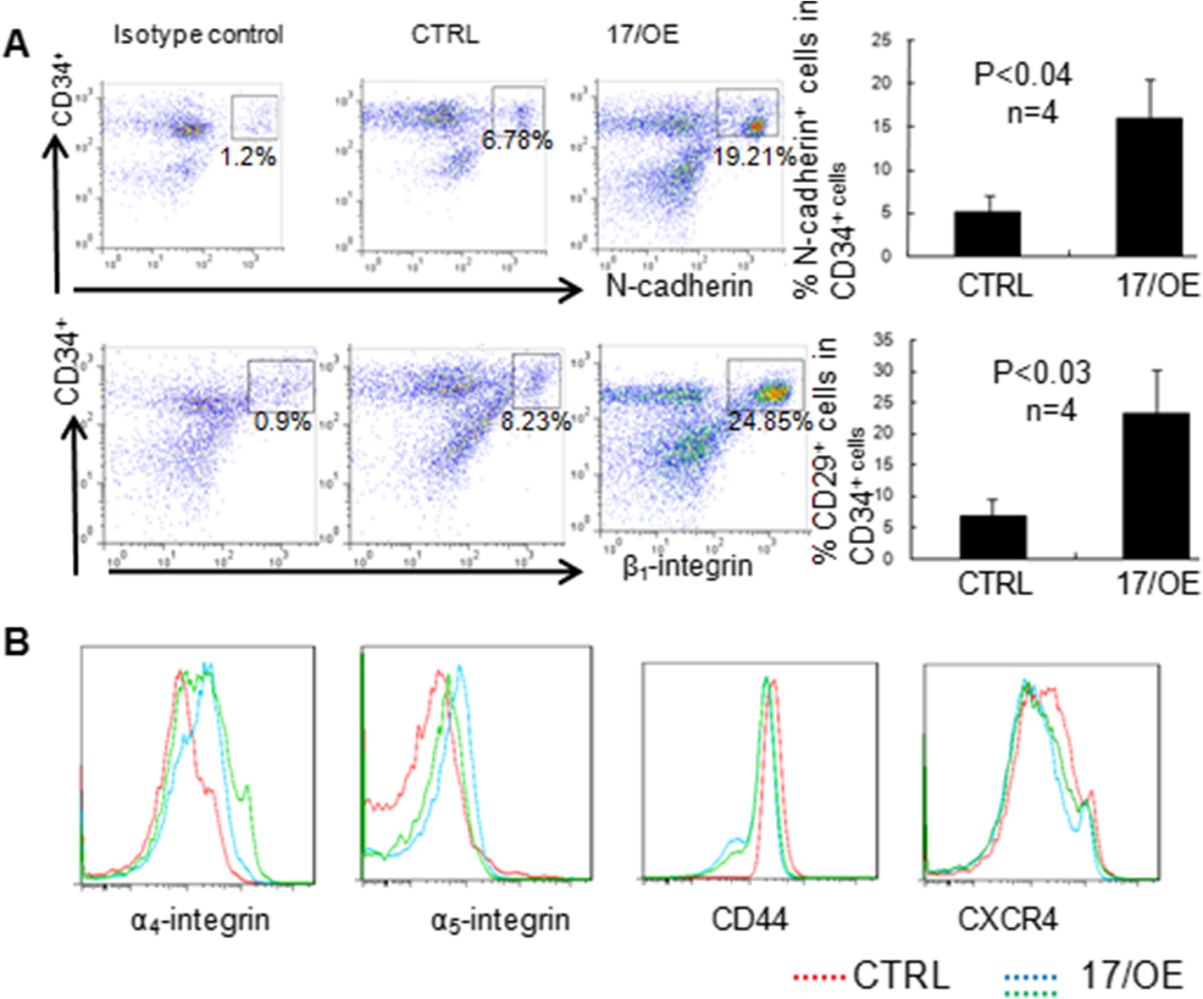
The adhesion molecule expression on CB CD34^+^ cells after *miR-17* overexpression. **a** The expression of N-cadherin and β_1_-integrin on CB CD34^+^ cells after *miR-17* overexpressing (17/OE) or control (CTRL) cells was analyzed by flow cytometry (*left panels*). The results are expressed as mean ± SD from multiple independent experiments (*right panels*). **b** Flow cytometry analysis of the expression of α_4_-integrin, α_5_-integrin, CD44 and CXCR4 on *miR-17* overexpressing CB CD34^+^ cells (*green* and *blue* lines) and CTRL CD34^+^ cells (*red* line)

Ectopic *miR-17* promotes the expansion of CB CD34^+^ cells in vitro but the hematopoietic reconstitution capability of 17/OE cells is reduced in vivo, which displays inconsistency. Except for expansion, the hematopoietic reconstitution capability of engrafted hematopoietic cells is also affected by the components from a physiological niche where HSCs reside in vivo. Successful hematopoietic reconstitution of human hematopoietic cells in engrafted mice needs proper proliferation, suitable attachment between HSCs and the bone marrow niche, as well as migration of HSCs in vivo. To examine whether the reduced hematopoietic reconstitution capability of CB CD34^+^ cells upon *miR-17* modulation in vivo is a result of improper attachment or migration in engrafted mice, we examined the expression patterns of selected adhesion and homing molecules known to be important for the hematopoietic reconstitution of human HSCs in the engrafted mice. As shown in Fig. 4a, 17/OE cells showed significantly higher expression levels of N-cadherin (3.16 ± 0.82-fold, n = 4) as well as β_1_-integrin (3.30 ± 0.98-fold, n = 4) compared to CTRL cells. We also analyzed the expression of α_4_ and α_5_ integrins in 17/OE cells, which were shown to be slightly increased. The expression levels of CD44 and CXCR4 were almost unchanged upon ectopic *miR-17* (Fig. 4b). The improper expression of N-cadherin and β_1_-integrin on CD34^+^ cells upon *miR-17* overexpression raised the possibility that the adhesion between 17/OE cells and their niche in vivo is regulated abnormally, which further leads to the reduced hematopoietic reconstitution capability of 17/OE cells in vivo. After knockdown of *miR-17*, there was a trend towards a decrease in the expression of β_1_-integrin and N-cadherin (Additional file 2: Figure S2) , although statistical analyses of the cohort indicated that it did not meet statistical significance (*p* > 0.05).

### Ectopic expression of *miR-17* alters CB CD34^+^ cell adhesion to hematopoietic niche components

**Fig. 5:**
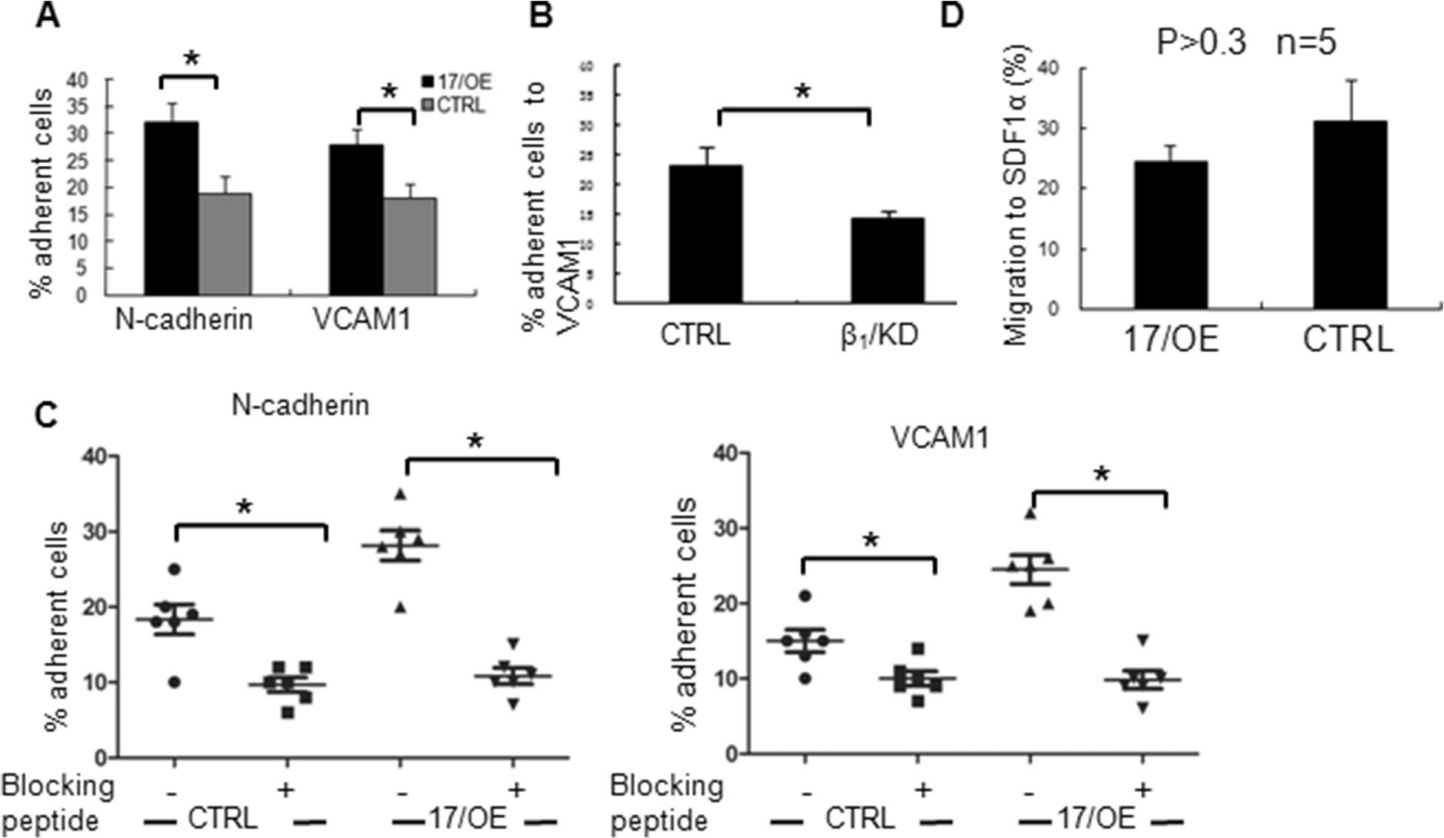
Effects of *miR-17* overexpression on adhesion and migration of CB CD34^+^ cells. **a** The adhesion of *miR-17* overexpressing (17/OE) CB CD34^+^ cells to N-cadherin or vascular cell adhesion molecule-1 (VCAM1) was significantly increased compared with that of control (CRTL) cells. Results are shown as mean + SD (n = 8 for N-cadherin and n = 10 for VCAM1). *p < 0.05, between 17/OE and CTRL groups (Student’s *t*-test). **b** The adhesion of 17/OE CD34^+^ cells with β_1_-integrin deficient (β_1_/KD) to VCAM1 was significantly decreased compared with that of CRTL cells. Mean + SD is shown (*p < 0.05, Student’s *t*-test). The CTRL is 17/OE CD34^+^ cells with scrambled shRNA. **c** The specific adhesion of CD34^+^ cells to N-cadherin or VCAM1 was confirmed by antibody blocking experiments. The adhesion was measured as described above. The adhesion of pre-treated cells was significant inhibited compared with that of non-treated cells (*p < 0.05, Student’s *t*-test). **d** The migration of 17/OE CB CD34^+^ cells towards SDF1α was slightly increased compared with that of CTRL cells, but statistical analyses indicated it did not meet statistical significance (*p* > 0.3, Student’s *t*-test)

Through adhesion to the corresponding components from niche in vivo, the adhesive moleculars expressed by hematopoietic cells regulated the interaction between hematopoietic cells and their niche. To examine whether the reduced hematopoietic reconstitution ability of 17/OE cells is related to the upregulated expression of N-cadherin and β_1_-integrin, we performed the adhesion assays in vitro. After incubation with the coated ligands, CB CD34^+^ cells showed a significant increase in the adhesion to N-cadherin or VCAM1 upon ectopic *miR-17* according to the CFC output (Fig. 5a). In order to identify whether the increase in adhesion potential of 17/OE cells to VCAM1 in vitro is directly from the altered expression levels of β_1_-integrin in 17/OE cells, we further knocked down the expression of β_1_-integrin in 17/OE cells with β_1_-integrin-specific shRNA (here called β_1_/KD; Fig. 5b). The adhesion potential of 17/OE CD34^+^ cells to VCAM1 was significantly blocked upon β_1_-integrin knockdown (Fig. 5b), which suggested that β_1_-integrin expressed on 17/OE CD34^+^ cells mediated, at least in part, the increase in interaction between 17/OE CD34^+^ cells and VCAM1 caused by ectopic *miR-17*. In order to demonstrate that the observed adhesion between 17/OE cells and N-cadherin or VCAM1 was directly mediated by their corresponding ligands, the cells were pre-incubated with soluble anti-N-cadherin peptide or anti-CD29 antibody, respectively, prior to performing adhesive assay. The adhesion potential was determined as described above. As shown in Fig. 5c, the specific adhesion of pre-incubated cells to each ligand was significantly reduced compared with that of nontreated cells. It is of note that the migration of *miR-17* overexpressed CB CD34^+^ cells towards SDF1α was slightly decreased compared to that of control cells. However, statistical analyses of this cohort indicated that it did not meet statistical significance (Fig. 5d), suggesting that the reduced hematopoietic reconstitution capability of *miR-17* overexpressed CB CD34^+^ cells in vivo does not result from the migration defect of transplanted 17/OE cells.

## Discussion

The self-renewal and differentiation of HSCs are controlled by the integration of cell-intrinsic and cell-extrinsic mechanisms, which still needs further research. Through directly or indirectly altering the expression levels of multiple genes, miRNAs become attractive candidates as HSC regulators. This study establishes that *miR-17* is differentially expressed in human CB hematopoietic cells (Fig. 1a), which indicates that *miR-17* may play distinct roles in hematopoietic cells at different developmental stages, although more CD markers are needed to further identify the subpopulations from CB CD34^+^ cells. In contrast to the more committed CB CD34^+^CD38^+^ cells, CB CD34^+^CD38^−^ populations express significantly higher levels of *miR-17*, suggesting that *miR-17* may be a key regulator during the development of HSCs. *miR-17* has been recognized either as an onco-miRNA or as a tumor suppressor depending on the cell type. The more precise subpopulations of human hematopoietic progenitors were established recently [29–31] so further studies are still needed to get a more detailed expression profile of *miR-17* in human CB HSCs based on the more precise hierarchy model.

By overexpression and knockdown studies, we demonstrated that *miR-17* regulates the growth of CB CD34^+^ cells and CD34^+^CD38^−^ cells in vitro. Ectopic expression of *miR-17* resulted in the promoted expansion of the phenotypic and functional CD34^+^CD38^−^ compartment in vitro. Although *miR-17* contains a highly conserved seed sequence between species and is expressed in hematopoietic cells at different stages from both mouse and human origin, there still exist different opinions concerning the function of *miR-17* on hematopoiesis [16, 21–23]. Our studies demonstrated that *miR-17* may specifically affect hematopoietic stem and early progenitor cells. This idea is evidenced by the fact that ectopic *miR-17* promotes the proliferation of CB CD34^+^CD38^−^ cells, especially within the first 15 days of culture when the cells are the least differentiated (Fig. 2b). The expanded hematopoietic cells upon ectopic *miR-17* can promote myeloid lineage fate (Fig. 2c), which is consistent with the results from Meenhuis group [19], demonstrating the conservative function of *miR-17* between mouse and human HSC to a certain extent. It seems that *miR-17* has almost no effect on the erythroid lineage development based on the lack of a significant difference between the number of BFU-E from 17/OE cells and that from the CTRL cells. The function of *miR-17* on human CB CD34^+^ cells is reinforced by knockdown studies on *miR-17*. Moreover, the expanded CB CD34^+^ cells by ectopic *miR-17* are capable of normal maturation ex vivo because they could differentiate into all of the lineages tested.

17/KD CB CD34^+^ cells may have a higher engraftment potential than CTRL CD34^+^ cells during the first 4 weeks after transplantation because PB MNCs from 17/KD recipients displayed a significantly higher percentage of human CD45^+^ populations at 4 weeks compared with that from the CTRL group (Fig. 3a, left panel). It is of great interest to note that the hematopoietic reconstitution potential of *miR-17* overexpressed CB CD34^+^ cells in vivo is reduced compared to that of CTRL CB CD34^+^ cells according to SCID repopulating cells assays (Fig. 3). *miR-17* was speculated to promote the hematopoietic reconstitution potential of CB CD34^+^ cells in vivo according to our in vitro assay. We observed no significant difference in the percentage of human erythroid cells, including CD45^−^CD36^+^ and CD36^−^GPA^+^ populations in the bone marrow of mice between the 17/OE and CTRL groups, which is consistent with the colony forming assay in vitro. However, a significantly lower percentage of human CD45^+^ cells and CD45^+^CD34^+^ cells was observed in the bone marrow of mice transplanted with 17/OE CD34^+^ cells compared to that in the bone marrow of mice transplanted with CTRL CD34^+^ cells. The levels of *miR-17* expression in GFP-positive cells from engrafted mice at 20 weeks were still up- or downmodulated (Fig. 3c), which indicated that this inconsistency between the in vivo and in vitro data was not due to the change of *miR-17* expression in vivo. Successful hematopoietic reconstitution of human HSCs in vivo requires suitable attachment between HSCs and the bone marrow niche, as well as migration between bones in engrafted mice. The mechanisms of engraftment have revealed a host of contributing factors, including adhesion molecules, such as integrins and CXCR4, as an important homing factor for HSCs [32–34]. According to our data, further studies are still needed to prove that the interaction between 17/OE cells and their niche cells in vivo is abnormal. However, compared to CTRL cells, 17/OE cells did express significantly higher levels of N-cadherin and β_1_-integrin (CD29) (Fig. 4), which helps mediate the binding of HSCs to bone marrow niche cells [35]. α_5_ (CD49e) and α_4_ (CD49d) integrins were also slightly upregulated. Both N-cadherin and CD29, together with other integrins and the chemokine receptor CXCR4, are known to be expressed in human HSCs and serve to promote their adhesion to the bone marrow niche cells [34, 36–38]. In support of this finding in vitro, a significant increase was observed in the adhesion of CB CD34^+^ cells to N-cadherin and VCAM1 following *miR-17* overexpression (Fig. 5a). This observation was further confirmed by knocking down the expression of β_1_-integrin in 17/OE CD34^+^ cells, which significantly reduced the adhesion potential of 17/OE CD34^+^ cells to VCAM1 (Fig. 5b). The decreased adhesion between β_1_/KD CB CD34^+^ cells and VCAM1 raised the possibility that the interaction between β_1_/KD CB CD34^+^ cells and their niche was not as strong as that of 17/OE CD34^+^ cells in vivo. It seemed that the significantly reduced hematopoietic reconstitution potential of *miR-17* overexpressed CB CD34^+^ cells in vivo is not a result of the defect of transplanted 17/OE cell migration between bones because the migration of *miR-17* overexpressed CB CD34^+^ cells in vitro towards SDF1α was only slightly increased compared to that of CTRL cells. All of these results indicated that interaction of *miR-17* overexpressed CB CD34^+^ cells with their niche may be abnormal in vivo and further prevent the efficient and continuous production of blood cells, which may be, at least in part, responsible for the reduced hematopoietic reconstitution potential of *miR-17* overexpressed CB CD34^+^ cells in vivo. The mechanisms underlying the enhanced expression of adhesive molecules in CB CD34^+^ cells upon ectopic *miR-17* are largely unclear and will be explored further in our laboratory.

## Conclusions

In summary, our data suggest the potential contribution of *miR-17* in the in vitro and in vivo function on human HSCs and HPCs. These data add adhesive molecules to the signaling network affected by *miR-17* and suggest a *miR-17*–N-cadherin (β_1_-integrin) pathway in human CB HSCs and HPCs. Therefore, further investigations of *miR-17* on hematopoiesis in vivo raises the possibility that *miR-17* may play a wider role in regulating hematopoietic development.

## Additional files

Additional file 1: Figure S1.
**(A)** The representative image of transfection efficiency (percentage of GFP-positive cells) was shown when sorted through FITC channel by FACS. The representative image of CFU-Mix **(B)**, CFU-GM **(C)** and BFU-E **(D)**. (TIFF 409 kb) Additional file 2: Figure S2.
**(A)** Flow cytometry analysis of the expression of GFP on CB CD34^+^CD38^−^/CD38^+^cells upon *miR-17* modulation after culturing for 20 days (red line) and control cells (black line). **(B)**. The expression of N-cadherin and β_1_-integrin on CB CD34^+^ cells after *miR-17* knockdown (17/KD) or control cells (CTRL) was analyzed by flow cytometry (*left panels*). The results are expressed as mean ± SD from multiple independent experiments (*right panels*). (TIFF 318 kb)

## Abbreviations

CB: Cord blood
CFC: Colony-forming cell
CFU: Colony-forming unit
CTRL: Control
GFP: Green fluorescent protein
HPC: Hematopoietic progenitor cell
HSC: Hematopoietic stem cell
miRNA: MicroRNA
MNC: Mononuclear cell
PB: Peripheral blood
PCR: Polymerase chain reaction
SD: Standard deviation
SDF1α: Stromal derived factor 1
VCAM1: Vascular cell adhesion molecule-1
