# Supplementary figures and images for: miR-17 promotes expansion and adhesion of human cord blood CD34+ cells in vitro
Source: Stem Cell Res Ther. 2015 Sep 7;6(1):168. doi: 10.1186/s13287-015-0159-1 (PMC4562375; doi:10.1186/s13287-015-0159-1)

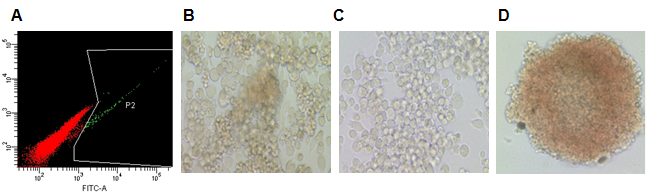

Supplement: Additional file 1: Figure S1. — (A) The representative image of transfection efficiency (percentage of GFP-positive cells) was shown when sorted through FITC channel by FACS. The representative image of CFU-Mix (B), CFU-GM (C) and BFU-E (D). (TIFF 409 kb) [file 13287_2015_159_MOESM1_ESM.tiff]

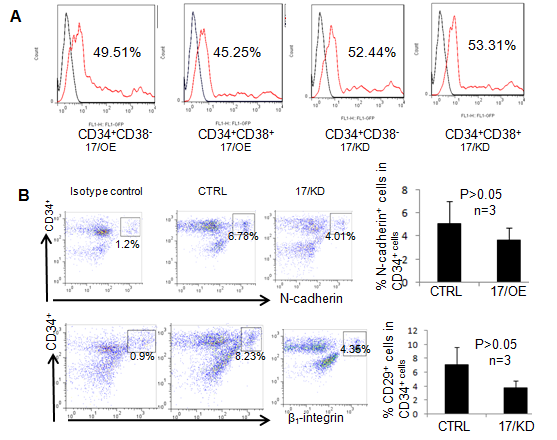

Supplement: Additional file 2: Figure S2. — (A) Flow cytometry analysis of the expression of GFP on CB CD34+CD38−/CD38+cells upon miR-17 modulation after culturing for 20 days (red line) and control cells (black line). (B). The expression of N-cadherin and β1-integrin on CB CD34+ cells after miR-17 knockdown (17/KD) or control cells (CTRL) was analyzed by flow cytometry (left panels). The results are expressed as mean ± SD from multiple independent experiments (right panels). (TIFF 318 kb) [file 13287_2015_159_MOESM2_ESM.tiff]
